# Retraction: Raf Kinase Inhibitor Protein (RKIP) Blocks Signal Transducer and Activator of Transcription 3 (STAT3) Activation in Breast and Prostate Cancer

**DOI:** 10.1371/journal.pone.0253350

**Published:** 2021-06-10

**Authors:** The PLOS ONE Editors

Following the publication of this article [1], concerns were raised regarding results presented in Figs 1, 2, and 4. Specifically,

- Vertical irregularities suggestive of splice lines were detected in the following panels:
  ○ Fig 1D, RKIP panel, between lanes 2 and 3.
  ○ Fig 2A DU145 RKIP panel, between lanes 1 and 2, as well as between lanes 2 and 3.
  ○ Fig 2A PC3 RKIP panel, between lanes 1 and 2, as well as between lanes 2 and 3.
- In Fig 2D, the PC3 PARP panel appears similar to the first two lanes of the right (DU145) panel, despite representing results obtained from different cell lines.
- In Fig 4A, the following similarities were detected between lanes presented in the middle panel and lanes presented in the right panel:
  ○ The first two lanes of the middle pY705 STAT3 panel appear similar to the first two lanes of the right pY705 STAT 3 panel. However, the corresponding Actin panel results for these lanes do not appear to match.
  ○ Lanes 3 and 4 in the middle panel appear similar to lanes 7 and 8 in the middle panel respectively, despite being used to represent different experimental conditions.
  ○ The last lane of the middle pY705 STAT3 panel appears similar to the last lane of the right pY705 STAT3 panel, despite being used to represent different experimental conditions.

The authors have not provided the original images underlying the panels of concern.

In light of the concerns affecting multiple figure panels that question the integrity of the data, the *PLOS ONE* Editors retract this article.

ELM agreed with the retraction and apologises for the issues with the published article. BB did not agree with the retraction. SY, MD, SC-K, KB, TL, KCY, FA-M, EC, and DC either did not respond directly or could not be reached.
